# Multidimensional analyses of the effect of exercise on women with depression: A meta-analysis

**DOI:** 10.1097/MD.0000000000026858

**Published:** 2021-08-20

**Authors:** Lin-Bo Yan, Jing-Zhi Zhang, Qian Zhou, Feng-Lin Peng

**Affiliations:** a Institutional affiliation: Guangxi Normal University; b College of Physical Education and Health, Guangxi Normal University, Guilin 541000, China.

**Keywords:** depression, exercise, meta-analysis, randomized controlled trial

## Abstract

**Background:**

The proportion of women is higher than men in depression. This is mainly due to women's physiological regulation is different from men, especially in puberty, menstruation, pregnancy, menopause, among others. Therefore, treating depressive women is still a health challenge. Besides, recent studies of exercise therapy have a more outstanding performance in treating depression, especially in contrast to drug therapy and psychotherapy. Its main advantages are convenience, quickness, no side effects, real-time, and long-term effectiveness.

**Objective:**

The aim of this study was to systematically review the clinical efficacy of exercise on women with depressive symptoms.

**Methods:**

Searching PubMed, The Cochrane Library, and Embase databases to collect randomized controlled trials about exercise in the treatment of depressive women. After literature screening, data extraction, and literature quality evaluation, the meta-analysis of acquirement data was performed with RevMan5.3 software.

**Results:**

A total of 2294 patients were included in 25 different articles totally. Meta-analysis shows that compared with the control group, exercise could relieve female depression (standard mean difference [95% confidence interval, CI] = −0.64 [−0.89 to −0.39], *Z* = 4.99, *P* < .001). Subgroup analysis shows that different types of exercise have significant effects in improving depression symptoms. Exercise therapy has better effect on depressive patients induced by physiology or disease than ordinary depressive patients.

**Conclusion:**

Exercise can significantly improve depressive symptoms in women.

## 1 Introduction

Depression is a common disease that manifests as a mental disorder. According to World Health Organization statistics, it is expected that depression will be the top leading cause of the global burden of disease in 2030.^[1]^ Nearly 800,000 people die from depression every year.^[2]^ During the onset of depression, the patient showed low mood, loss of interest, and lack of energy. At the same time, it is accompanied by anxiety, sleep disturbance, loss of appetite, self-guilt, inattention, and the depressed symptom that is not a direct effect of a medical condition. Not only psychological, but also social and physiological factors affect depression in different aspects. People with tremendous stress and dysfunction in life are more likely to suffer from depression. However, a series of diseases such as cardiovascular disease, diabetes, and cancer are also important causes of depression.

At present, the clinical treatments of depression are still focused on psychology and antidepressants. However, neither psychotherapy nor medication can meet the patient's urgent need for recovery. Other factors may interfere with the results during the long treatment period.^[3]^ Therefore, more and more researchers focus on non-drug treatments and realize that changes in living behavior can help depressive patients recover. Numbers of studies have shown that exercise has a significant impact on depression, but it differs depending on exercise types. A meta-analysis^[4]^ showed that exercise as a treatment has a moderate to significant effect on depressive patients. Moreover, some researches^[5–10]^ show that yoga, Qigong, and Tai Chi can improve the symptoms of depression, but the effects of above methods are related to the coach's training level and exercise duration. In most cases, exercise as adjuvant therapy and other interventions (dietary regulation, drug therapy, among others) have a more remarkable effect. However, this is just a comparison between exercise as monotherapy and conventional care. When comparing to psychotherapy or medication, there is almost no difference in effect.

The prevalence of depression in women is higher than that in men, probably related to women's unique menstrual cycle and hormone secretion.^[11,12]^ For women, depression often occurs around the regular onset of the luteal phase in the menstrual cycle. The inducing factors for women with depression are mainly changes in metabolism, sex, lifestyle, ovarian activity during adolescence, pregnancy, and menopause, accompanied by complex mood disorders.^[13–15]^ Besides, women are susceptible to breast cancer and ovarian syndrome, which can also cause depression and anxiety.^[16]^

The direction of previous meta-analysis researches usually focuses on the classification of exercise. The study by Kvam et al^[4]^ analyzed the effects of exercise and non-intervention, psychotherapy, and medication by randomized controlled trials (RCTs) of different interventions. Schuch et al^[17]^ selected different exercise types, such as moderate-intensity, aerobic, and high-intensity exercise. And part of the meta-analysis has subdivided the research objects, based on age, prenatal, postnatal, breast cancer, menopause, and so on. The meta-analysis of Gong et al^[18]^ compared yoga as an intervention treatment with a control group (prenatal care, prenatal exercise, social support, among others), and the results support the efficacy of yoga on depression in pregnant women. Although Nakamura et al^[19]^ partially confirmed the improvement of physical activity on depression in prenatal and postpartum women, the analysis results observed unexplainable heterogeneity. This may be related to the history of depression or the subject's mental activity. However, there is no summary of exercise for women with special conditions (prenatal, postpartum, menopause, breast cancer, ovarian syndrome, and so on) and depressive women without exceptional circumstances in the database.

Considering both the increasing influence of depression and the lack of knowledge in the database, it is necessary to update the content about the effect of exercises on depression in women. The present meta-analysis objective is to investigate the effect of exercise as a treatment for depression in women. We will examine the positive effect of different exercise and the effects of different types of depression in women (prenatal, postpartum, menopause, breast cancer, among others). We will also assess the effect of exercise on depression in different types of women.

## 2 Materials and methods

### 2.1 Search

A search of PubMed, The Cochrane Library, and Embass database for RCTs on exercise on depression in women published from 2005 to the present were conducted on October 15, 2020. We also searched to retrieve all potential relevant unpublished reported materials and conference proceedings referred to the topic. We set the search time limit of nearly 15 years and limit the diagnostic criteria to control heterogeneity. Search terms include Depression, Depressive disorder, Exercise, Physical activity, Women, Female, Randomly, Randomized controlled trial, controlled clinical trial, and all types of exercise (eg, walking, running, aerobic exercise, resistance exercise, strength exercise, yoga, Qigong, Tai Chi).

### 2.2 Study selection

We selected all RCTs in which the language was English, and the study was exercising intervention on depression in women. There are 2 standards for participants: All adults aged >18 years with a diagnosis of depression as defined by the Diagnostic and Statistical Manual of Mental Disorders, Fourth Edition (DSM-IV), International Statistical Classification of Diseases and Related Health Problems 10^th^ Revision (ICD-10) criteria or a confirmed depression scale; the participants limited to women. We exclude studies with men or a mixture of men and women and include articles of depression by diseases and physiology. Because depression often occurs in adults, our age limit is ≥18 years, and the ages of patients included in the exhaustive articles are 18 to 64 years. Besides, we include at least 2 groups of the articles. The experimental group was given exercise intervention, and the control group was given routine intervention measures like daily activities or lower-intensity exercise. We include documents that meet the above criteria and rule out those that meet the following criteria: intervention subjects are non-exercise conditions, and not only women; non-English articles; no experimental data can be calculated; experimental group is a combined intervention study; repeated research.

### 2.3 Outcome measures

We selected the following 4 related depression scale surveys: The Center for Epidemiological Studies Depression Scale (CES-D),^[20]^ Beck Depression Inventory (BDI), The Edinburgh postnatal depression scale (EPDS), and The Hospital Anxiety, and Depression Scale (HADS). These are widely used on a mainstream scale, with a wide range of applications.

### 2.4 Literature retrieval

Two researchers conducted 3 rounds of screening participants obtained from the preliminary search in an independent double-blind manner. The study did not meet the inclusion criteria or did not meet the quality requirements was excluded. Data extraction is performed using a unified feature table. The primary information includes the name of the first author, time, region, number of subjects, course of the disease, age, the primary intervention measure, evaluation indicators, and follow-up time. The 2 researchers then checked the results with each other. If there is a disagreement, the third researcher will arbitrate.

### 2.5 Effect estimators’ level of evidence

We use the bias risk assessment tool recommended by the *Cochrane Collaboration* to evaluate the research bias risk. There are 7 items: random sequence generation, allocation concealment, blinding of participants and personnel, blinding of outcome assessment, incomplete outcome data, selective reporting, and other biases. These 7 items are mainly evaluated from 5 aspects: the selection and implementation of the experimental plan, the measurement of the result data, the completion of the subjects, and whether the result report is objective and complete.

### 2.6 Data analysis

We use RevMan5.3 software for meta-analysis. Because the measurement data are continuous variables, the mean difference is used as the effect indicator (confidence interval is 95%). There is no significant difference in the baseline, and the endpoint after the intervention (M ± SD) is used as the main effect parameter. When we use the *Q* test, if *P* > .10, it shows no significant heterogeneity; in the *I*^*2*^ test, if *I*^*2*^ < 50%, it shows no significant heterogeneity. When there is no significant heterogeneity among the studies, the results should be analyzed using the fixed-effects model; for the studies with significant heterogeneity, the results should be analyzed using the random-effects model. Without affecting the authenticity of the analysis results, we select subgroup analysis according to factor classification to explore the source of its heterogeneity.

### 2.7 Additional information

With regard to ethical reviews, meta-analysis is based on data from published studies that do not involve patients and therefore do not require.

## 3 Results

### 3.1 Study selection

**Figure 1:**
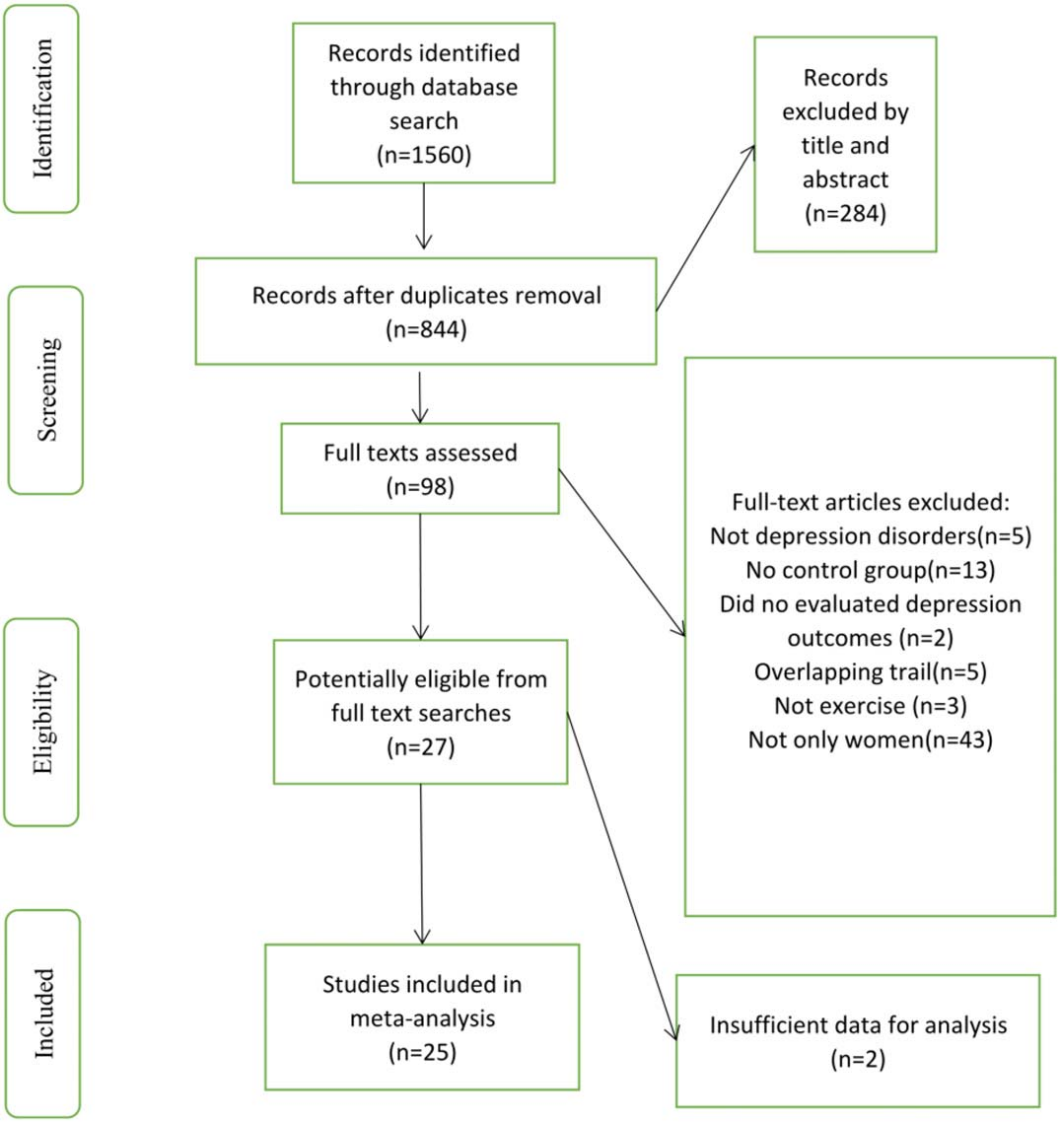
Literature selection flow diagram.

We collected 1560 articles through preliminary search and then eliminated duplicate publications. Determining the research content by reading the titles and abstracts, we screened out 284 documents. After reading the complete text, we screened 98 articles based on their experimental design and output indicators. According to the inclusion criteria and quality evaluation, 25 articles were selected for qualitative analysis, and all of which^[21–46]^ were finally determined, with 2294 participants. We conducted a meta-analysis on these documents. The database search was completed on October 2020. Figure 1 displays the research procedure and the flow of the study selection and inclusion.

### 3.2 Study characteristics and individual studies’ results

Twenty-five studies were included in the qualitative and quantitative sustainability analysis. Their characteristics and main results are displayed in Table 1. For each of the studies included, methodological aspects, participants’ characteristics, and key results are displayed. Overall, 2294 participants with depression were included.

**Table 1:** Information extraction from Articles.

Study	Participants	N	Age	Conditions	Exercise	Duration	Main outcome
Aibar et al, 2019^[21]^	Outpatients recruited through e-mail and telephone after contacting 2 associations of post menopausal women	55/52	69.98 (7.83)/ 66.79 (10.14)	1)Pilates 2) maintain daily habits	2 sessions/wk 60 min warm-up (10 min) Main pilates training (35 min) Cool-down (10 min)	12	HADS
Elizabeth 2006^[22]^	Healthy sedentary postmenopausal women	9/8	56 (4)	1) Aerobic exercise 2) Non-exercise	5 Sessions/wk 30 min 50% HRR	6	HADS
Razazian et al, 2016^[23]^	Female patients diagnosed with MS were recruited from the MS center of Hospital	18/18	33.33 (7.40)/33.11 (6.60)	1) Hatha Yoga 2) Non-exercise	3 Sessions/wk 60 min Hatha Yoga	7	BDI
Callaghan et al, 2011^[24]^	Female inpatients	19/19	55.0 (9.9)/50. 4 (15.2)	1) Treadmill aerobic exercise (referred intensity) 2) Treadmill aerobic exercise (prescribed intensity)	3 Sessions/wk referred intensity, chosen exertion level, was established using the RPE scale	4	BDI
Chattha et al, 2008^[25]^	Perimenopausal outpatients recruited through advertisements	54/54	49 (3.6)/48 (4. 00)	1) IAYT 2) easy body movements	5 Sessions/wk 60 min	8	PSS
Ho et al, 2017^[26]^	Breast cancer patients undergoing adjuvant radiotherapy	63/58	49.1 ± 7.8/49.8 ± 8.4	1) DMT 2)radiotherapy and standard nursing care	2 Sessions/wk 90 min	3	PSS
Chu et al, 2015^[27]^	Volunteers recruited through flyers and word of mouth from a university and the surrounding community	26/26	27.58 ± 6.12/24.58 ± 5.17	1) Yoga 2) Maintain usual level of physical activity	2 Sessions/wk 60 min Pranayama, asana, and savasana	8	PSS, CES-D
Mohammadi et al, 2015^[28]^	Pregnant women at 26–32 wks pregnancy	36/36	25.5 ± 4.6/25.3 ± 5.2	1) Low-intensity stretching and breathing practices 2)No intervention	3 Sessions/wk 60 min	8	EPDS
Coll et al, 2018^[29]^	Pregnant women between 16 and 20 wks	192/387	27.2 ± 5.5/27.3 ± 5.5	1)Aerobics and floor Exercise 2)No intervention	3 Sessions/wk 60 min	16	EPDS
Daley et al, 2015^[30]^	Women who presented with depression within 6 mon of giving birth	41/38	31.7 ± 5.3/29.3 ± 5.7	1) facilitated exercise 2) usual-care	3 Sessions/wk (wks 1–12) 3–5 Sessions/wk (wks 13–24) 30 min moderate-intensity	24	EPDS
Field et al, 2012^[31]^	Clinically depressed pregnant women recruited from two prenatal ultrasound clinics affiliated	46/46	24.4 ± 4.7/26.0 ± 5.6	1) Tai chi/yoga 2) waitlist	1 Session/wk 20 min	12	CES-D
Robledo-Colonia et al, 2012^[32]^	Pregnant women between 16 and 20 wks of gestation, with a live fetus at the routine	37/37	21 ± 3/21 ± 3	1) aerobic exercise 2) no intervention	3 Sessions/wk 60 min 55%–75% of maximal heart rate	12	CES-D
van der Waerden et al, 2013^[33]^	Ultrasound scan women recruited through	46/48	43.06 ± 8.88/43.87 ± 7.67	1) Aerobic exercise 2) waiting-list	Low to moderate intensity	8	CES-D, PSS
Cho et al, 2012^[34]^	Advertisements females who were ≥18 y of age with a confirmed diagnosis of breast, colorectal, or ovarian cancer	46/56	49.76 ± 8.81/50.53 ± 10.24	1)aerobic exercise 2) no intervention	3 Sessions/wk 20 min Moderate-intensity	16	CES-D
Lanctot et al, 2016^[35]^	Breast cancer patients receiving chemotherapy	58/43	51.23 ± 9.29/50.29 ± 9.54	1) BYP-BC 2) waiting period	8 Sessions/wk 90 min 2 Prayanamas, shavasanas, and psychoeducational themes	8	BDI
Chu et al, 2009^[36]^	Women with mild to moderate depressive symptoms recruited from a university community	18/18	26.4 ± 7.3/24.6 ± 4.8	1) aerobic exercise 2) stretching exercise	1 Session/wk 30–40 min 40%–55% of VO2R (wks 1–3) 65%–75% of VO2R (wks 4–10)	10	BDI
Zkan et al, 2019^[37]^	Postpartum women who had a spontaneous vaginal delivery in a maternity hospital	34/31	28.90 ± 4.83	1) exercise program 2) standard care pratices	5 Sessions/wk 30 Min mild- and medium-level exercises (wks 1–2) Medium-level exercises (wk 3) severe-level exercises (wk 4)	4	EPDS
Ko et al, 2008^[38]^	Women recruited froma maternity center	31/30	34.17 ± 3.20/34.33 ± 3.53	1)Pilate/Yoga/music 2)no intervention	3 Sessions/wk 60 min	3	CES-D
Mutrie et al, 2018^[39]^	Women with polycystic ovary syndrome	22/24	30.2 ± 5.1/28.8 ± 6.0	1) intermittent aerobic training 2) no training	3 Sessions/wk 30–50 min	16	HADS
Nanette 2007^[40]^	Women during appointments at outpatient clinics for chemotherapy or radiotherapy	82/92	51.3 ± 10.3/51.8 ± 8.7	1) Aerobic exercise Program 2) usual care	3 Sessions/wk 45 min Warm-up (5–10 min) Exercise (20 min) Cool-down relaxation	12	BDI
Amanda 2008^[41]^	Women with postnatal depression	16/15	≤21	1) Moderate-intensity aerobic exercise 2) usual care	105 min/wk Moderate-intensity	12	EPDS
Satyapriya et al, 2013^[42]^	Women in wk 12 of gestation	51/45	26.41 ± 3.01/24.96 ± 2.58	1) SWEP 2) no exercise	3 Sessions/wk 60 min Warm-up, aerobic element exercise, stretching, and relaxation	16	HADS
Samira 2018^[43]^	Women between 12th to 20th wks of gestation	23/22	53.4 ± 8.6/51.3 ± 8.7	1) IAYT 2) standard antenatal stretching practices	7 Sessions/wk 60 min	16	BDI
Davis et al, 2016^[44]^	Women with type 2 diabetes and depression	15/14	53.3 ± 6.0/53.6 ± 8.4	1) Behavioral activation exercise 2) usual care	2 Sessions/wk 60 min Walking, Zumba, Pilates, step aerobics, cardio-kickboxing, and power Yoga	12	BDI
Kyle 2015^[45]^	Pregnant women with elevated depression or anxiety symptoms	23/23	29.74 ± 5.40/30.57 ± 4.46	1) Yoga 2) treatment as usual	1 session/wk 75 min	8	EPDS

BYP-BC = Bali Yoga Program for Breast Cancer Patients; Conditions: IAYT = integrated approach to yoga therapy, DMT = dance movement therapy; Exercise: HRR = heart rate reserve; Participants: MS = Multiple Sclerosis, SWEP = study of water exercise during pregnancy, VO2R = oxygen uptake reserve. BDI = Beck Depression Inventory, CES-D = The Center for Epidemiological Studies Depression Scale, EPDS = The Edinburgh postnatal depression scale, HADS = The Hospital Anxiety and Depression Scale, PSS = The Perceived Stress Scale.

### 3.3 Assessment of publication bias

**Figure 2:**
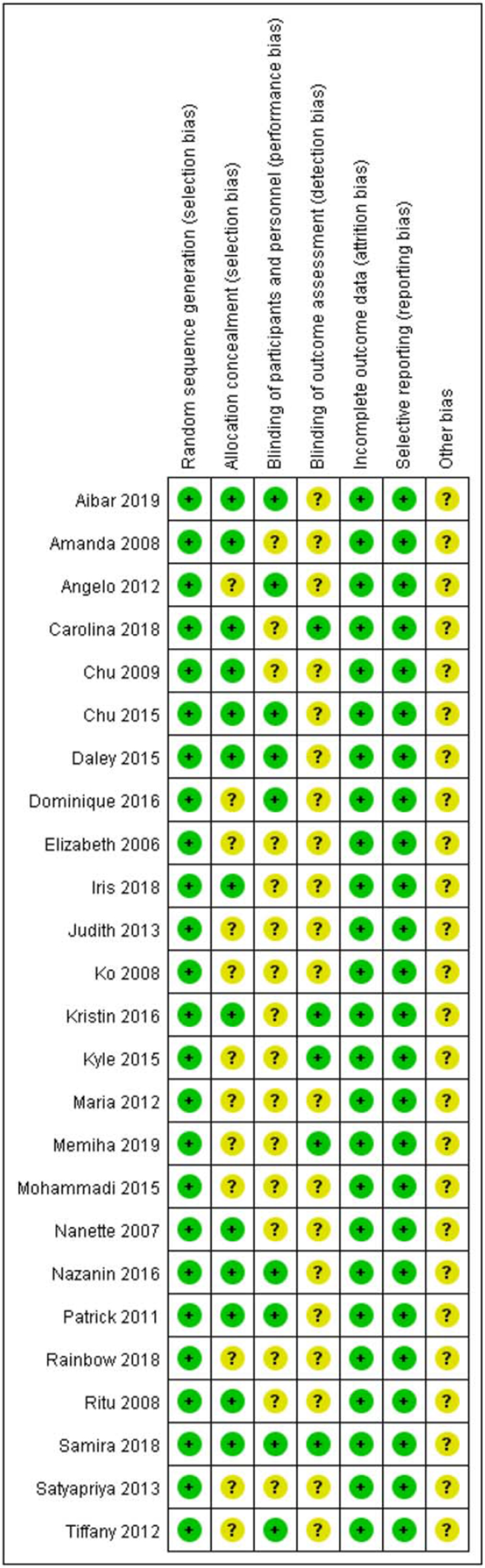
Risk of bias summary: judgments about each risk of bias item for each included trail.

**Figure 3:**
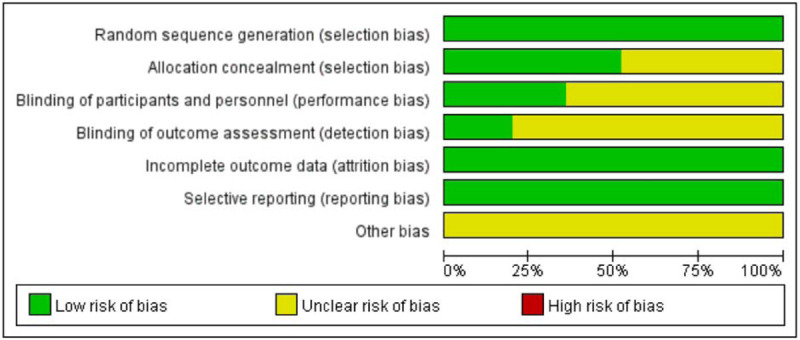
Risk of bias graph: judgments about each risk of bias item presented across all included trials.

Twenty-five studies mention the use of random allocation schemes, 13 articles detail the hiding methods of specific allocation schemes, 9 articles specify the implementation process of blinding, and 5 article illustrates the implementation of blinding results evaluation. All the 25 articles ensured that the experimental results were all omitted, and there was no selective reporting phenomenon. Both the study quality and risk of bias ratings are displayed in Figure 2 and Figure 3.

### 3.4 Meta-analysis results

#### 3.4.1 Analysis 1: exercise versus control

**Figure 4:**
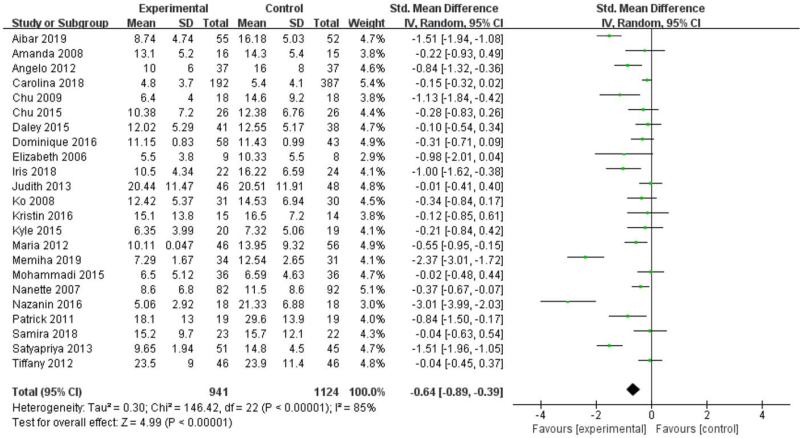
Forest plot of meta-analysis on the effect size of exercise on standard mean difference on depression in women.

Based on 23 studies (2065 participants), we found that reduction in depressive symptoms after treatment showed a significant effect in favor of exercise (Fig. 4), (standard mean difference [SMD] [95% confidence interval, CI] = −0.64 [−0.89 to −0.39], *Z* = 4.99, *P* < .001); the heterogeneity between studies was significant and high (*I*^*2*^ = 85%).

#### 3.4.2 Risk of bias across studies

**Figure 5:**
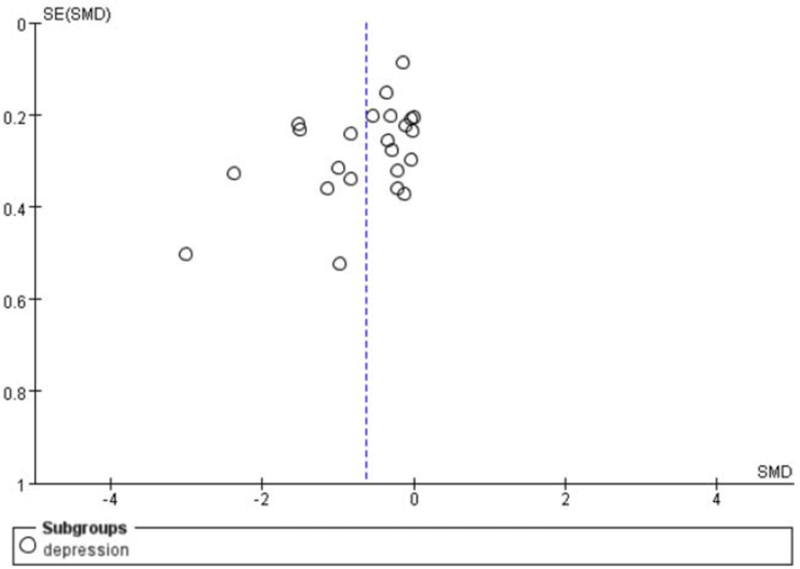
Funnel plot of all studies included. Each first sustainability SMD and their belonging SE are plotted. SE = standard error, SMD = standard mean difference.

The risk of bias across studies is by means of a funnel plot (Fig. 5). It reveals a low risk of publication bias.

### 3.5 Subgroup analysis

#### 3.5.1 Exercise versus control

We performed 2 subgroup analyses to compare studies according to the exercise mode and the patient's condition.

**Figure 6:**
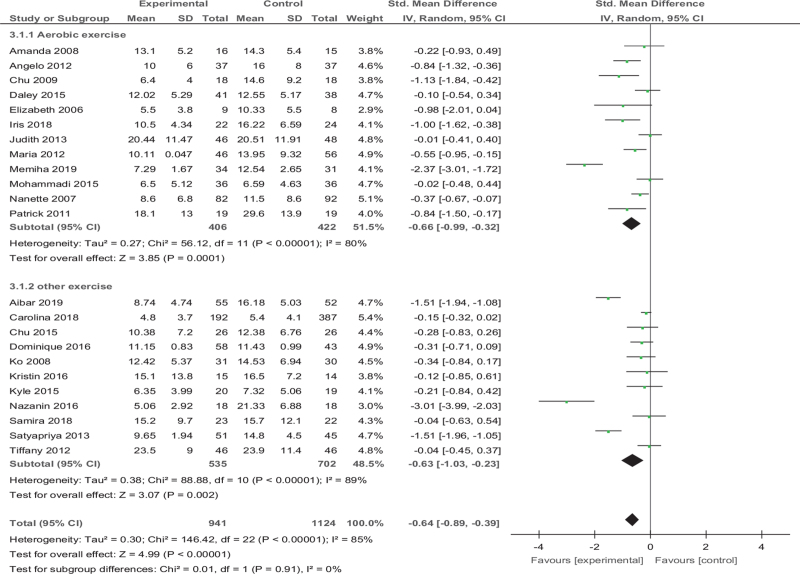
Forest plot of subgroup analysis on the effect size of exercise mode on standard mean difference on depression in women. CI = confidence interval, SD = standard deviation.

In the first subgroup analysis (Fig. 6), we divide the studies into aerobic exercise groups (12 studies, 828 participants) and other exercise groups (11 studies, 1237 participants) according to the exercise mode of the intervention group. We choose random-effect model combination, aerobic group (SMD [95% CI] = −0.66 [−0.99 to −0.32], *Z* = 3.85, *P* < .001) and other exercise group (SMD [95% CI] =−0.63 [−1.03 to −0.23], *Z* = 3.07, *P* = .002) were both significant. The difference in effect size between the subgroups was not significant (*P* = .91).

**Figure 7:**
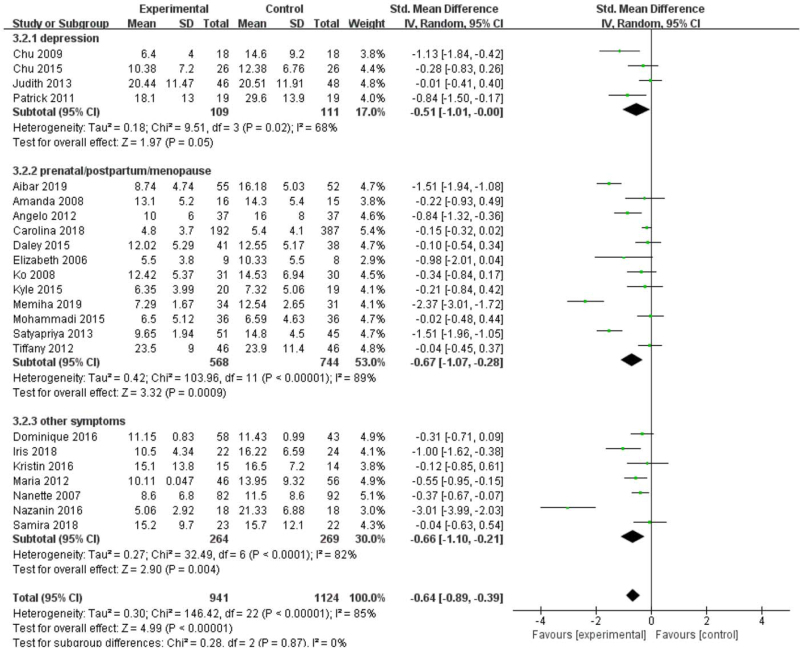
Forest plot of subgroup analysis on the effect size of patient's own condition on standard mean difference on depression in women. CI = confidence interval, SD = standard deviation.

In the second subgroup analysis (Fig. 7), we divide the study into depression group (4 studies, 220 participants) according to the patient's own condition, prenatal/postpartum/menopause group (12 studies, 1312 participants) and other symptoms group (7 studies, 533 participants). We choose random-effect model combination, depression group (SMD [95% CI] = −0.51 [−1.01 to −0.00], *Z* = 1.97, *P* = .05), prenatal/postpartum/menopause group (SMD [95% CI] = −0.67 [−1.07 to −0.28], *Z* = 3.32, *P* < .001] and other symptoms group (SMD [95% CI] = −0.66 [−1.10 to −0.21], *Z* = 2.90, *P* = .004) all showed a remarkable effect. The difference in effect size between the subgroups was not significant (*P* = .87).

#### 3.5.2 Analysis 2: exercise versus CES-D

**Figure 8:**
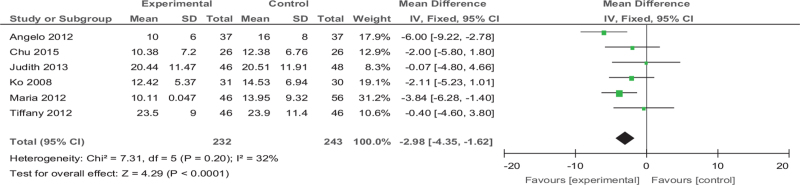
Forest plot of meta-analysis on the effect size of exercise on The Center for Epidemiological Studies Depression Scale on depression in women. CI = confidence interval, SD = standard deviation.

Among the 25 articles we finally included, 5 articles^[27,31,32,33,34,38]^ reported on CES-D, a total of 475 patients. The results showed that the exercise group's CES-D was significantly lower than the control group (MD [95% CI] = −2.98 [−4.35 to −1.62], *Z* = 4.29, *P* < .001, *I*^*2*^ = 32%), and the difference was statistically significant as we showed in Figure 8.

#### 3.5.3 Analysis 3: exercise versus The Perceived Stress Scale

**Figure 9:**
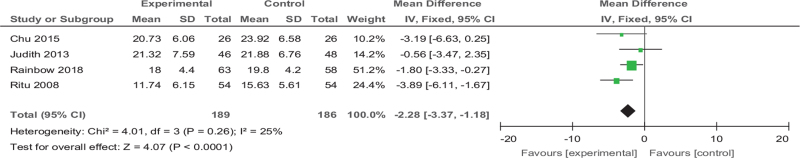
Forest Plot of meta-analysis on the effect size of exercise on The Perceived Stress Scale on depression in women. CI = confidence interval, SD = standard deviation.

Among the 25 articles, we finally included, 4 articles^[25,26,27,33]^ reported on The Perceived Stress Scale (PSS), a total of 375 patients. The results showed that the PSS of the exercise group was significantly lower than the control group (MD [95% CI] =−2.28 [−3.37 to −1.18], *Z* = 4.07, *P* < .001, *I*^*2*^ = 25%), and the difference was statistically significant as we showed in Figure 9.

#### 3.5.4 Analysis 4: exercise versus EPDS

**Figure 10:**
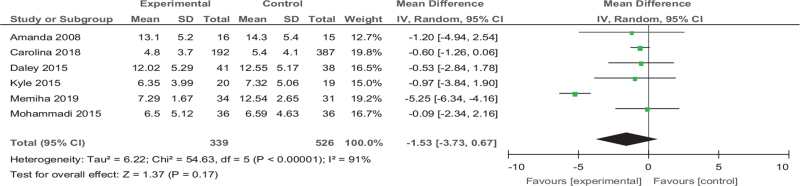
Forest plot of meta-analysis on the effect size of exercise on The Edinburgh postnatal depression scale on depression in women. CI = confidence interval, SD = standard deviation.

Among the 25 articles, 6 articles^[28,29,30,37,42,46]^ are reported on EPDS, a total of 865 patients. The results showed that the EPDS of the exercise group was non-significantly than the control group (MD [95% CI] =−1.53 [−3.73 to 0.67], *Z* = 1.37, *P* = .17, *I*^*2*^ = 91%) as we showed in Figure 10.

#### 3.5.5 Analysis 5: exercise versus HADS

**Figure 11:**
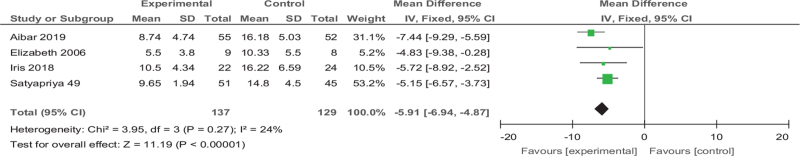
Forest plot of meta-analysis on the effect size of exercise on The Hospital Anxiety and Depression Scale on depression in women. CI = confidence interval, SD = standard deviation.

Among the 25 articles, we finally included 4 articles^[21,22,40,43]^ reported on HADS, a total of 266 patients. The results showed that the HADS of the exercise group was significantly lower than the control group (MD [95% CI] = −5.91 [−6.94 to −4.87], *Z* = 11.19, *P* < .001, *I*^*2*^ = 24%), and the difference was statistically significant as we showed in Figure 11.

#### 3.5.6 Analysis 6: exercise versus BDI

**Figure 12:**
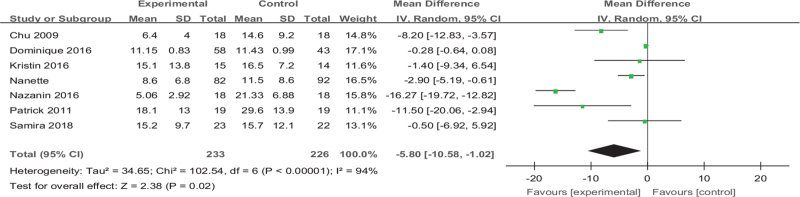
Forest plot of meta-analysis on the effect size of exercise on Beck Depression Inventory on depression in women. CI = confidence interval, SD = standard deviation.

Among the 25 articles, 7 articles^[23,24,35,36,41,44,45]^ reported on BDI, a total of 459 patients. The results showed that the BDI of the exercise group was significantly lower than the control group (MD [95% CI] =−5.80 [−10.58 to −1.02], *Z* = 2.38, *P* = .02, *I*^*2*^ = 94%), and the difference in effect between groups was significant as we showed in Figure 12.

#### 3.5.7 Subgroup analysis 2: exercise versus EPDS

**Figure 13:**
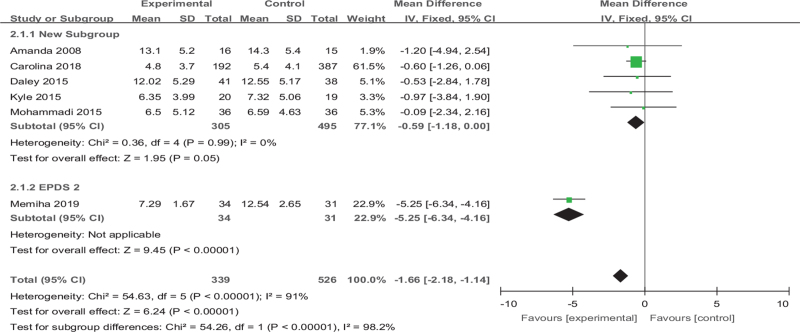
Forest plot of subgroup analysis on the effect size of exercise on The Edinburgh postnatal depression scale on depression in women. CI = confidence interval, SD = standard deviation.

The 6 articles we included have a high heterogeneity (*I*^*2*^ = 91%), and the source may be related to exercise duration. In the 6 articles, except for the *Memiha 2019* intervention 4 weeks, other articles have been intervention for >8 weeks, so we divide these articles into 2 groups according to the length of the duration for subgroup analysis. There is no heterogeneity among the 5 articles of Group1, and the results have significant differences (MD [95% CI] = −0.59 [−1.18 to −0.00], *Z* = 1.95, *P* = .05, *I*^*2*^ = 0%); Group 2 has only one article (MD [95% CI] = −5.25 [−6.34 to −4.16], *Z* = 9.45, *P* < .001) (Fig. 13).

#### 3.5.8 Subgroup analysis 3: exercise versus BDI

**Figure 14:**
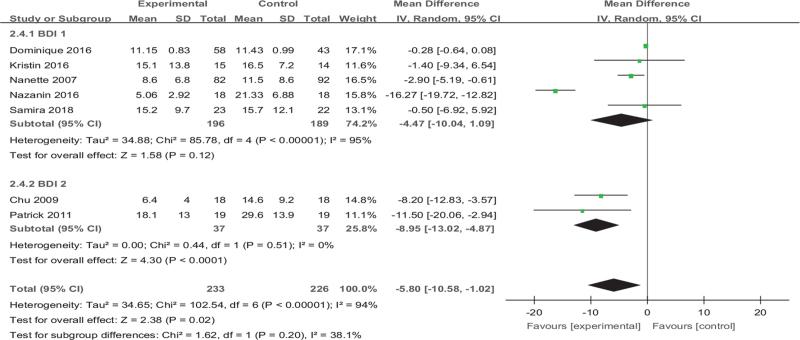
Forest plot of subgroup analysis on the effect size of exercise on The Edinburgh postnatal depression scale on depression in women. CI = confidence interval, SD = standard deviation.

The 7 articles we included have highly heterogeneous (*I*^*2*^ = 91%), and the source may be related to the symptoms on depression in women. Only 2 of the 7 articles (Chu et al, 2009^[36]^, Callaghan et al, 2011^[24]^) were ordinary depression in women, and the other 5 articles were all suffering from other diseases (multiple sclerosis, breast cancer, primary Sjogren syndrome, diabetes). Therefore, according to whether they have other diseases, they are divided into 2 groups for subgroup analysis. There is high heterogeneity among the 5 articles in Group 1, and the difference in effect was significant (MD [95% CI] = −4.47 [−10.04 to −1.09], *Z* = 1.58, *P* = .12, *I*^*2*^ = 95%). There is no heterogeneity among the articles of Group 2, and the difference in effect was significantly (MD [95% CI] =−8.95 [−13.02 to −4.87], *Z* = 4.30, *P* < .001, *I*^*2*^ = 0%) (Fig. 14).

## 4 Discussion

The main purpose of our study is to analyze the efficacy of exercise therapy for depression in women. The study showed exercise treatment, as a significant monotherapy, can effectively reduce the symptoms in the treatment of women with depression. Mood states rely on endorphin secretion. Exercise augments endorphin secretion which, in turn, reduces depression levels.^[47]^ Also exercise may alleviate depression through neuromolecular mechanisms.^[48]^ For women, depression often occurs during the active ovarian period. Adolescence, menstruation, pregnancy, and menopause are all “window of vulnerability” for depression,^[49]^ but they may resist this period from a psychological level. As a long-term treatment plan for patients to follow, on the one hand, it effectively alleviates depressive symptoms to patients living discomfort. However, it can prevent psychological stress due to examination. Accordingly, compared with pharmacotherapy or psychotherapy of depression, exercise has similar beneficial effects.

Nevertheless, our analysis 1 shows the exercise has a significant effect on reducing depression in women (*P* < .001), but there is still high heterogeneity (*I*^*2*^ = 85%). We guess that there are 2 reasons for this situation: different types of exercise will have different degrees of effect on depressive patients; the state of the patients may affect the therapeutic effect. We immediately subgroup analysis found that different types of exercise (aerobic exercise, resistance exercise, yoga, among others) have significant effects on depression in women, which is consistent with Kvam et al,^[4]^ Cramer et al.^[50]^ However, it is significant for the treatment of depressive patients with physiological conditions or other diseases (breast cancer, multiple sclerosis, primary Sjogren syndrome, and so on). It may be because exercise slightly improves the quality of life or reduce stress for ordinary depressive patients. But exercise can reduce the pain (dysmenorrhea, chemotherapy, among others) caused by physiological or disease conditions and indirectly improve depressive patients. The effect of exercise remains to be investigated because we included a small number of RCTs (only 4 articles) in ordinary depressive patients.

In addition, we will classify the different scales included in the articles and analyze them separately to examine the practicality of exercise therapy in more detail. We found that CED-S, HADS, BDI, EPDS statistics show that the treatment effect of exercise in depressive women is more prominent, and EPDS, BDI results are highly heterogeneous. We guess that it is related to the scales’ own evaluation criteria and experimental conditions (exercise duration or research subjects).

We conducted a subgroup analysis of all the articles that included EPDS, and the results show that the degree of improvement in depressive patients is related to the exercise duration. However, due to the limited number of included articles, the findings remain high heterogeneity. We suspect that the heterogeneity and the personal recovery progress of postpartum women about the reasons, the latter may also need evidence. EPDS applies only to postpartum women. The degree of pain during childbirth itself will inevitably affect the post-treatment. But either because of the subjects or intervention itself or scale limitations, the movement of the efficacy of depression in women is still relatively significant.

Finally, we conduct a subgroup analysis of the articles whose indicator is BDI. The results show that although the exercise has some effect, there are specific differences in the symptoms of patients with primary depressive mood and with disease complicated by depressed mood. It is similar to the results of a meta-analysis for breast cancer patients.^[51]^ Although the final result is that exercise has a slight improvement in the depression of breast cancer patients, the number of references is limited, and the specific effects need to be studied in depth. From the intrinsic quality of the scale, BDI has high sensitivity. It is the final result that may fluctuate as a wide range of data appears. Wang et al^[51]^ also conducted the meta-analysis on the application of BDI and found that BDI has a certain sensitivity. The questions it raised is too transparent for self-tested people, and it is more suitable for some patients who voluntarily participate or are familiar with their own conditions.

Interestingly, we additionally found that some articles selected PSS to perform stress tests on patients. We also carried out a PSS analysis that showed a significant improvement in exercise for the role of patient pressure. Life stress is one of the risk factors for depression. Nowadays, many people choose to exercise as a decompression project in their free time.

## 5 Strengths and limitations

Our study included not just ordinary depression in women, but also through its contrast with depression by the disease or physiological problems (pregnancy, postpartum, menopause) caused analyzed and discussed, and provided new ideas for the treatment of depressive by exercise. We also analyzed the therapeutic effect of different types of exercise, and evaluated the effects of different scales on the treatment of depressive patients. At the same time, we also recognize several limitations. If we take all the experiment blindly for depression, it may cause psychological resistance, making it difficult to conduct and affecting the results. The assessment of depression scales is generally an indicator of subjective impressions. We also found that for the RCTs of depressive patients, although randomized, it is difficult to ensure that baseline indicators of each group are similar. This situation will cause high heterogeneity, making it difficult for us to judge the difference in efficacy by comparing endpoints.

## 6 Conclusions

The existing meta-analysis usually shows that the exercise intervention group reflects a more significant treatment effect than the control group. Therefore, exercise is an effective means to treat the women with depression, regardless of the cause including puberty, pregnancy, postpartum, menopause, or disease. However, due to the lack of research and publication of the highest quality deviation method, it is difficult to explain the findings. For now, the actual effect of exercise therapy in female patients with clinical depression requires further study in the future of more high-quality articles.

## Acknowledgments

The authors are grateful to Yu-hu Lv, PhD, and Wen-hua Huang, PhD, for proofreading this manuscript.

## Author contributions

**Conceptualization:** Lin-Bo Yan, Feng-Lin Peng

**Data curation:** Lin-Bo Yan, Jing-Zhi Zhang, Qian Zhou, Feng-Lin Peng

**Formal analysis:** Lin-Bo Yan, Qian Zhou, Feng-Lin Peng

**Investigation:** Lin-Bo Yan, Jing-Zhi Zhang, Qian Zhou, Feng-Lin Peng

**Methodology:** Lin-Bo Yan, Jing-Zhi Zhang, Qian Zhou, Feng-Lin Peng

**Project administration:** Lin-Bo Yan.

**Resources:** Lin-Bo Yan, Qian Zhou, Feng-Lin Peng

**Software:** Lin-Bo Yan, Jing-Zhi Zhang, Qian Zhou, Feng-Lin Peng

**Supervision:** Lin-Bo Yan, Feng-Lin Peng

**Validation:** Lin-Bo Yan, Jing-Zhi Zhang, Qian Zhou, Feng-Lin Peng

**Visualization:** Lin-Bo Yan, Jing-Zhi Zhang, Qian Zhou, Feng-Lin Peng

**Writing – original draft:** Lin-Bo Yan.

**Writing – review & editing:** Lin-Bo Yan.
